# Editorial: Nanoparticle Vaccines Against Infectious Diseases

**DOI:** 10.3389/fimmu.2019.02615

**Published:** 2019-11-07

**Authors:** Rajko Reljic, África González-Fernández

**Affiliations:** ^1^Institute for Infection and Immunity, St. George's, University of London, London, United Kingdom; ^2^Centro de Investigaciones Biomédicas (CINBIO) (Centro Singular de Investigación de Galicia), Universidad de Vigo, Vigo, Spain; ^3^Instituto de Investigación Sanitaria Galicia Sur (IIS-GS), Vigo, Spain

**Keywords:** nanoparticles, vaccine, liposomes, immunity, infection

Vaccines remain the most realistic means of controlling, eliminating, and eventually completely eradicating an infectious disease. Many vaccines have been successfully implemented over the course of recent medical history and some have succeeded in completely (small pox) or near-completely eradicating (polio) their target diseases. However, many human pathogens are still in need of an effective vaccine, including viruses such as human immunodeficiency (HIV), herpes simplex (HSV), respiratory syncytial (RSV), Chikungunya, Dengue, Ebola, and Hepatitis C; bacteria such as *Mycobacterium tuberculosis* or *Salmonella dysenteriae* and parasites such as *Plasmodium* spp., to name a few.

Challenges in developing an effective vaccine against these infectious diseases are multiple, ranging from antigenic variability (HIV), multiple genotypes or serotypes (Norovirus, Dengue, Influenza), complex pathogenesis or life cycle of pathogen (tuberculosis, malaria), lack of correlates of protection (TB) and finally, the paucity of the adjuvants and delivery systems suitable for human application. With regards to the latter, it is true to say that non-live, subunit vaccine development has been historically severely hampered by safety issues associated with the adjuvants and delivery systems.

Nanoparticles (NPs) have emerged as a promising approach for vaccine delivery as both antigen delivery platform and immunomodulators. Their use in vaccine candidates from early preclinical to late stage clinical testing is a testament to their success as a promising, new approach, alongside the more conventional protein plus adjuvant, viral vector, or attenuated whole organism vaccine approaches. There are many different types of NPs, both synthetic and natural, as well as further varieties that are not considered classically as NPs, but are physically and functionally closely related to them, such as liposomes, virus-like particles, bacterial spores, and immunostimulating complexes.

The ability of NPs to interact with immune components and to induce humoral and cellular immune responses make them particularly amenable for vaccine design. Furthermore, they have been successfully applied by different routes (systemic and throughout the mucosa), and have been demonstrated capable of modifying and broadening of the immune profiles. They can increase antigen stability (time, temperature, proteolysis) and confer substantial flexibility to vaccine formulation, allowing the incorporation of diverse antigens and immunostimulants, compared to conventional subunit/adjuvant vaccines.

Many of these advantages are highlighted in this Research Topic but it is also necessary to draw attention to the areas for further improvements, including a better understanding of the mechanisms of NPs vaccine immunogenicity and a more efficient antigen presentation by the molecular histocompatibility complex molecules, especially the so called cross-presentation by class I molecules performed by specialized dendritic cells, following the endocytic/phagocytic uptake of NPs. Also, and very importantly, NPs design and technology may need to be further improved to allow for an “off-the-shelf” approach to their application and testing rather than the complex and time/resource demanding process of customized production.

In this Research Topic, we include several articles that focus on NP-based vaccine delivery against infectious diseases and also review articles that summarize and critically assess the progress that has been achieved so far in the specific areas of NP vaccine development. It is hoped that the presented evidence will deepen our understanding of their mode of action and the overall potential of NPs for translation of this vaccine approach to human application.

Thus, Kim et al. reported a novel method of generating bio-designed NPs utilizing a bacterial expression system and the capacity of RNA molecules to act as chaperones. Using Middle East respiratory syndrome-coronavirus (MERS-CoV) as the infection target, they demonstrated that NPs can be assembled in the expression host and that this was entirely dependent on chaperoning capacity of RNA since mutations in the RNA-binding domain abolished formation of NPs. The resulting NPs were immunogenic in mice and induced blocking antibodies against MERS virus. This approach of generating protein-only based NPs could be further optimized (i.e., in terms of yields and homogeneity) and developed as a generic NPs platform against other infectious diseases.

In another article, the utility of mucosally delivered chitosan-based vaccine against swine influenza A virus (SwIAV) was demonstrated by Dhakal et al., who reported that strong cross-reactive mucosal IgA and cellular immune responses in the respiratory tract were induced in young piglets using this vaccination approach. Intranasal delivery of these NPs loaded with SwIAV antigens resulted in a reduced nasal viral shedding and lung virus titres in pigs, suggesting that this vaccination approach could offer a broader coverage than the current attenuated strain-specific SwIAV.

Chitosan was also tested, alongside arginine-rich protamine (PR) and polyarginine (PARG)-based NPs in a more mechanistic in our own studies (Peleteiro et al.), reporting that PR and PARG showed a superior immunomodulating ability, as measured by enhanced reactive oxygen species production, activation of the complement cascade, cytokine production, and mitogen-activated protein (MAP) kinases/nuclear factor κB activation. When complexed with recombinant Hepatitis B glycoprotein, and compared against each other, protamine-based NPs elicited higher IgG levels than PARG NPs.

The utility of NPs as a delivery system for immunododulators and adjuvants rather than antigens was illustrated in the article by Takahashi et al. They showed that the biodegradable carbonate apatite (CA)-based nanoparticles can serve as the delivery system for the CpG oligodeoxynucleotide (CpG ODN) adjuvant and that this combination was more potent in activating dendritic cells and induced more diverse cytokine profiles than CpG ODN alone. When used with a model antigen, the NPs/CpG ODN induced higher level humoral and cellular immune responses in mice, and in particular enhanced CD8+ T cell responses, suggesting that this vaccination approach is particularly suitable for viral infections which require cytotoxic T cells alongside the neutralizing antibodies to control the viremia.

Another example of the benefit of combining NPs with adjuvants was provided also in the study by Malik et al., who showed that an anthrax antigen when combined with trimethyl-chitosan nanoparticles (TMC-PA) and either CpG ODN or polyinosinic: polycytidylic acid (Poly I:C) adjuvant induced higher immune responses in mice than any other combination in that vaccine formulation. In a different approach, Wagner-Muniz et al. reported that laboratory-generated polyanhydride nanoparticles based on 1,8-bis-(p-carboxyphenoxy)-3,6-dioxaoctane (CPTEG), 1,6-bis-(p-carboxyphenoxy)hexane (CPH) and sebacic acid (SA), could be employed as an effective vaccination strategy against *Streptococcus pneumoniae*, with strong immune responses and good-level of protection after the challenge of mice with the bacteria.

Finally, in our own studies (Copland et al.), we demonstrated that mucosal delivery of a mycobacterial poly-antigen coated onto heat-inactivated *Bacillus subtilis* spores (“Spore-FP1”) induced systemic and mucosal immune responses in mice, characterized with elevated antigen-specific IgG and IgA titres in the serum and bronchoalveolar lavage, antigen-specific memory T-cell proliferation in both CD4^+^ and CD8^+^ compartments, and resident memory T cells accumulation in the lungs. When used to boost the current TB vaccine, BCG, this vaccine candidate provided superior protection in mice challenged with aerosolised *M. tuberculosis*.

In addition, the collection also includes two review articles on lipid-based particles as a highly versatile vaccine delivery system. Thus, Corthesy and Bioley reviewed the potential of liposomes and liposomes derivatives as mucosal vaccine delivery systems, while Nisini et al. performed a critical assessment regarding the application of liposomes in a broader context of infectious diseases. Gao et al. provided an extensive review of the current state of virus-like particles (VLP) as an emerging and highly attractive vaccine delivery system, focusing in particular on the use of VLP in the context of HIV infection. And finally, Pati et al. reviewed the current state of NPs-based vaccines against infectious diseases, highlighting the key challenges and the potential for further progress.

The promising results obtained with several type of NPs highlight the potential of this vaccine approach for the development of new vaccines in the near future. However, further studies are still required to address in greater detail the issues regarding their safety, immunogenicity, stability, cost, scaling-up potential, and the use of appropriate animal models and clinical assays in humans. Even so, the significant body of evidence already generated, as partly illustrated in this Research Topic, underscores the translational potential of NPs in vaccine research and development, not only in the context of infectious diseases but potentially also other conditions such as autoimmune diseases and cancer.

## Author Contributions

All authors listed have made a substantial, direct and intellectual contribution to the work, and approved it for publication.

### Conflict of Interest

The authors declare that the research was conducted in the absence of any commercial or financial relationships that could be construed as a potential conflict of interest.

